# Partial Molar Solvation Volume of the Hydrated Electron Simulated Via DFT

**DOI:** 10.1021/acs.jpcb.3c05091

**Published:** 2024-02-29

**Authors:** William R. Borrelli, Kenneth J. Mei, Sanghyun J. Park, Benjamin J. Schwartz

**Affiliations:** Department of Chemistry and Biochemistry, University of California, Los Angeles, Los Angeles, California 90095-1569, United States

## Abstract

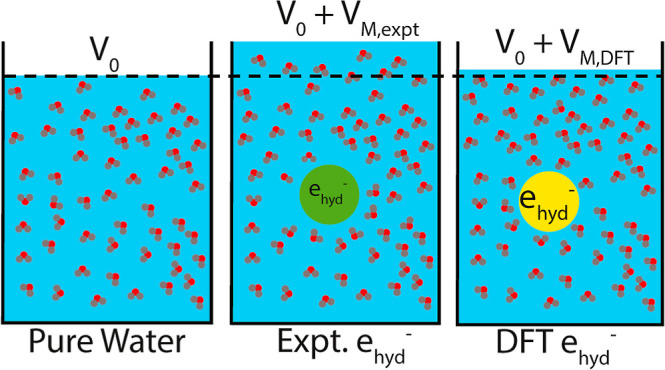

Different simulation models of the hydrated electron produce different solvation structures, but it has been challenging to determine which simulated solvation structure, if any, is the most comparable to experiment. In a recent work, Neupane et al. [*J. Phys. Chem. B***2023**, 127, 5941–5947] showed using Kirkwood–Buff theory that the partial molar volume of the hydrated electron, which is known experimentally, can be readily computed from an integral over the simulated electron–water radial distribution function. This provides a sensitive way to directly compare the hydration structure of different simulation models of the hydrated electron with experiment. Here, we compute the partial molar volume of an ab-initio-simulated hydrated electron model based on density-functional theory (DFT) with a hybrid functional at different simulated system sizes. We find that the partial molar volume of the DFT-simulated hydrated electron is not converged with respect to the system size for simulations with up to 128 waters. We show that even at the largest simulation sizes, the partial molar volume of DFT-simulated hydrated electrons is underestimated by a factor of 2 with respect to experiment, and at the standard 64-water size commonly used in the literature, DFT-based simulations underestimate the experimental solvation volume by a factor of ∼3.5. An extrapolation to larger box sizes does predict the experimental partial molar volume correctly; however, larger system sizes than those explored here are currently intractable without the use of machine-learned potentials. These results bring into question what aspects of the predicted hydrated electron radial distribution function, as calculated by DFT-based simulations with the PBEh-D3 functional, deviate from the true solvation structure.

## 1 Introduction

When an excess electron is injected into liquid water, a stable species known as the hydrated electron (e_hyd_^–^) is formed. Although the hydrated electron is nominally the simplest chemical solute, different simulation models have produced a wide variety of possible solvation structures for this object, making it challenging to directly connect simulations with experiment. A good simulation model of the hydrated electron should be able to correctly predict its absorption spectrum,^1^ temperature dependence,^2−4^ resonance Raman spectrum,^4,5^ time-resolved photoelectron spectroscopy,^6^ and the way the hydrated electron behaves in ionic solutions.^7−10^ Another important point of contact between simulation and experiment is the hydrated electron’s molar solvation volume, *V*_M_, which has been determined experimentally to be 26 ± 6 cm^3^/mol.^11,12^ Since *V*_M_ is directly related to the hydrated electron’s solvation structure, it is important that any good simulation model expand the volume of the water solution in which the e_hyd_^–^ resides by this amount.

In the past several decades, one-electron mixed quantum-classical (MQC) molecular dynamics (MD) simulations have been the workhorse for modeling the dynamics of solvated electron systems. The quantum mechanical treatment of the single excess electron allows for efficient solution of the one-electron Schrödinger equation, while classical treatment of the solvent permits simulations with many hundreds of solvent molecules for times up to nanoseconds. For MQC simulations, the interaction between the excess electron and solvent is accounted for using a pseudopotential, and for hydrated electrons in particular, several different pseudopotentials have been presented.^13−17^ There is an extensive literature investigating the performance of different MQC-based e_hyd_^–^ models, each of which produces a unique hydration structure;^2,9,18,19^ to date, no single MQC model has been able to reproduce all of the various experimental observables listed above. We note that our group has previously advocated for a noncavity model^16^ of the hydrated electron, but a recent work^20^ has suggested that a cavity model is closer to the correct structure. However, not all cavity models are equal, and more work needs to be done to understand which cavity-forming model(s) of the hydrated electron, if any, is the most correct.

In just the past few years, the availability of more powerful computational resources has allowed access to ab initio MD simulations of solution-phase molecular systems; for systems like the e_hyd_^–^, the only affordable electronic structure method accessible for such systems is density-functional theory (DFT),^7,21−23^ although multi-timestep algorithms involving DFT and MP2^24,25^ and extensions via machine learning potentials^26,27^ have been attempted. The idea is that with a higher level of theory that does not rely on the empirical parameters or assumptions inherent with pseudopotentials, DFT-based hydrated electron simulations should produce more accurate structures and dynamics compared to MQC. However, the system sizes and time scales with which one can simulate hydrated electrons via fully ab initio DFT are far smaller and shorter than those routinely used in MQC-MD studies, casting doubt on whether or not the solvation structure of the hydrated electron is converged with respect to the number of waters being simulated.^23^

With that said, we note that DFT-based simulations have provided new insights in comparison to MQC models. For example, DFT has correctly captured the trend of the temperature dependence of the absorption spectrum due to destructuring of the first solvent shell;^27^ however, the absolute value of the absorption maximum in those simulations was off by 400 meV relative to experiment. We also showed that the resulting spectra were not only blue-shifted relative to experiment but also had the incorrect shape.^23^ In addition, our previous work found that DFT predicts inhomogeneous broadening^28^ and a red-shift of the absorption spectrum in the presence of Na^+^,^29^ results that are the opposite of what is observed experimentally. DFT simulations do capture the mixing of the hydrated electron’s density into the antibonding orbitals of coordinating waters,^30^ a result is consistent with experimental X-ray absorption experiments,^31^ something that clearly cannot be accounted for with MQC simulations. However, the bearing that this mixing of the electron density into the first-shell waters has on the correctness of the predicted solvation structure has not yet been established.

We also note that DFT-based simulations investigating the binding energies of hydrated electrons^19,32^ have elucidated that these species either do not reside at the air/water interface, or that electrons at the air/water interface have the same binding energy as those in the bulk, which is also consistent with experiment.^33,34^ In addition, DFT studies have correctly reproduced the resonance Raman spectrum of the hydrated electron,^4,5,25^ although MQC structures, which are qualitatively different from DFT-generated structures, can also sometimes reproduce this observable, depending on the level of theory employed.^4,5^

To date, most of the DFT-based simulations of the hydrated electron have employed GGA^35^ (BLYP) or hybrid (PBEh-D3) exchange–correlation functionals.^7,22,23,29,36^ Some groups also have employed QM/MM approaches,^21,37,38^ which allow access to longer simulation time scales and larger box sizes, but such simulations still have limitations as to the number of waters one can treat quantum mechanically and also require additional approximations for treating how waters diffuse into and out of the QM region. The standard DFT-based hydrated electron simulation size in the literature to date is 64 quantum mechanical waters,^22,24,36^ although we have recently extended such simulations to 128 fully quantum mechanical waters and tens of ps of simulation time after equilibration.^23^ We also have argued that even if such calculations were fully converged with respect to system size and simulation time, DFT may not provide a high enough level of theory to accurately capture the structure of an excess electron that primarily resides between the water molecules.^7,23,29^

Recently, Neupane et al.^20^ used a method introduced by Schnell et al.^39^ and refined by Krüger and Vlugt^40,41^ for calculating the partial molar volume of the hydrated electron from the simulated radial distribution function (RDF) using the Kirkwood–Buff (K–B) approach.^42,43^ Their method not only makes it much simpler to calculate the molar solvation volume than the way our group did this in the past,^15,19^ but it also allows a straightforward interpretation of various contributions of the electron’s solvation structure to the partial molar volume. Neupane et al.^20^ calculated *V*_M_ for several pseudopotential-based MQC models of the e_hyd_^–^,^13,15,16^ and they also attempted to estimate the partial molar volume using our previously published DFT-based ab initio simulations,^23^ although they were unable to do so quantitatively.

Here, we take advantage of the K–B method used by Neupane et al.^20^ to present a rigorous quantitative calculation of the partial molar volume of the hydrated electron simulated via DFT with a hybrid functional as a function of system size. Since this method of calculating *V*_M_ directly depends on the e_hyd_^–^-water radial distribution function, this methodology provides the only connection to date between the simulated solvation structure and an experimental observable. We recognize that solvation structure involves a variety of interrelated properties such as the RDF, coordination numbers, coordination distances, angular distributions, and so on, which we have investigated for the hydrated electron in past work.^23,29^ All the conclusions that we make about the hydrated electron’s solvation structure in this work come directly from the simulated RDF, which encodes many of the aforementioned properties.

We find that at the standard 64-water simulation size used by most groups,^22,24,36^ the predicted *V*_M_ of the DFT-based hydrated electron is only 7.5 cm^3^/mol, roughly one-third of the experimentally measured number. For our 128-water simulations,^23^ which are currently the largest presented in the literature, the solvation volume is only 12.9 cm^3^/mol, about half of what is seen experimentally. We also calculate the size dependence of each solvent structure contribution (cavity, first shell, second shell, and third shell) to the hydrated electron’s partial molar volume. These results suggest that there are aspects of the DFT-predicted solvation structure that are incorrect, as we have argued in previous work;^7,29^ however, the degree to which, and which parts of, the structure disagrees from experiment remains to be seen.

The DFT simulations performed in this work use the hybrid PBEh-D3 exchange–correlation functional, which has 25% Hartree–Fock exchange as is common for such functionals in the literature.^44^ We note that other groups have used this same functional but with a higher fraction (40%) of Hartree–Fock exchange,^22,36^ or used the PBE0 hybrid functional without dispersion correction,^22^ and others have used the PBEW1-D3/MP2 multistep method^24^ to simulate the hydrated electron, but all of these different simulations obtained a similar radial distribution function as that obtained here within error.^23^ This means that these other simulations also significantly underestimate the molar solvation volume of the hydrated electron, suggesting that this is a problem with the currently available simulation sizes, the use of DFT with exchange–correlation functionals that are not optimal for this system, or some combination of both.

## 2 Methods

To extend the work of Neupane et al.^20^ to DFT-based ab initio models of the hydrated electron, we used our previously published simulations with 47, 64, and 128 waters to investigate the system size dependence of the contributions to the e_hyd_^–^’s partial molar volume.^23^ The details of these simulations are outlined in ref (23). Briefly, we simulated DFT-based hydrated electron MD trajectories using the CP2K package. Simulations were done in the *N*, *V*, *T* ensemble at a temperature of 298 K with a time step of 0.5 fs. A Nose–Hoover chain thermostat^45^ was coupled to the system in order to maintain constant temperature, and the volume of the simulation cell was chosen to reproduce the experimental bulk water density at 298 K and 1 atm pressure. We used the PBEh exchange–correlation functional along with Grimme’s DFT-D3 correction (PBEh-D3)^46,47^ and a triple-ζ basis set. The calculation of Hartree–Fock exchange^44^ was expedited by the use of the auxiliary density matrix method.^48^

It is important to note that our simulations were done at constant volume as opposed to at constant pressure, which may have an impact on the calculation of the partial molar volume. Each simulation was run for at least 20 ps after equilibration, which makes them among the longest trajectories of the ab initio DFT-hydrated electron presented in the literature so far. It is worth noting, however, that *V*_M_’s calculated from the K–B formalism are very sensitive to subtleties in the radial distribution functions (RDFs), which may not be fully converged with equilibrated trajectories with durations of only a few tens of ps.

To calculate the partial molar volume of the hydrated electron from our MD simulations, we followed the K–B formalism introduced by Schnell et al.,^39^ refined by Krüger and Vlugt,^40,41^ and used by Neupane et al.^20^ Given the K–B integral expression for an open system
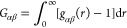
1where *g*_αβ_(*r*) is the center-of-mass RDF for a solute, denoted α, relative to a solvent, denoted β. The partial molar volume can be subsequently calculated via^20^
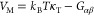
2where *k*_B_ is Boltzmann’s constant, *T* is the temperature in kelvin, and κ_T_ is the isothermal compressibility of the solvent. Here, we use the isothermal compressibility calculated for liquid water simulated via DFT with the PBEh-D3 level of theory,^49^ and we explore the use of other values of κ_T_ for calculating *V*_M_ in the Supporting Information.

To account for the finite nature of the closed simulation system, we use the following weighting function introduced by Krüger
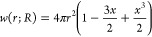
3with *x* = *r*/*R*. With the accompanying weighting function, the finite-sized K–B integral is as follows^20^
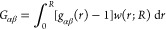
4where *R* is taken as half the simulation cell length.

Because we were concerned about the convergence of the K–B integrals for the small system sizes and trajectory lengths available from DFT-based simulations, we performed an additional analysis exploring the dependence of the *V*_M_ on system size using MD simulations of a classical chloride ion in water, as described in detail in the Supporting Information. The chloride ion has a qualitatively similar RDF as the DFT-simulated hydrated electron, and a similar *V*_M_ as the (experimental) hydrated electron, providing an excellent test of the accuracy of the K–B formalism when applied to simulations with the small sizes and shorter trajectories characteristic of AIMD. We find that even at the 47-water size, the K–B predicted *V*_M_ of chloride matches that of full-scale simulations within 10%, and at the 128-water size with a ∼20 ps trajectory, the predicted *V*_M_ essentially agrees with the “correct” value within error. This suggests that the partial molar volumes that we predict from our DFT simulations of the hydrated electron are indeed reasonable estimates of the converged value.

## 3 Results and Discussion

By calculating the partial molar volume of the DFT-simulated hydrated electron at three different system sizes, our goal is to answer two fundamental questions. First, is the DFT-simulated hydrated electron’s partial molar volume and thus solvation structure converged with respect to the number of waters included in the simulation cell? Second, does the use of DFT to simulate the e_hyd_^–^ more accurately reproduce the experimental partial molar volume compared to pseudopotential-based MQC methods?

**Figure 1:**
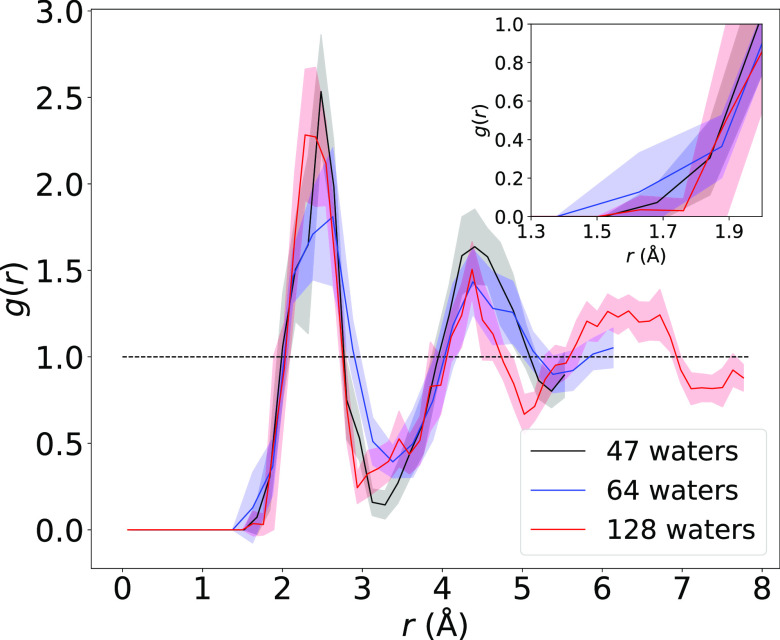
Electron center-of-mass to water center-of-mass RDFs for the DFT-simulated hydrated electron with 47 (black), 64 (blue), and 128 (red) waters, with simulation details given in ref (23). The magnitudes of the highly structured solvation shell peaks and their positions show a significant dependence on system size, indicating that the DFT-generated solvation structure is not converged with respect to system size, even with 128 waters. The inset shows the same data magnified in the region where the RDF first begins to rise, which defines the central cavity. Details of how the error bars were calculated and how the RDFs depend on the chosen bin size are given in the Supporting Information.

To answer the first question, we begin by examining DFT-simulated electron center-of-mass to water center-of-mass RDFs for each system size, plotted in Figure 1, with error bars computed as twice the standard error of the mean from block averaging,^50^ that is, the 95% confidence interval, which is consistent with the way that we have reported error bars for DFT-based simulations of this object in the past^23^ (see the Supporting Information for details on the way the uncertainties were computed). We note that in our fully periodic simulations the size of the simulation box increases along with the increasing number of quantum mechanical water molecules. Both the changing cell size and the changing number of quantum-mechanically treated waters constitute finite-size effects that could impact the value of the calculated *V*_M_. Unlike in our previous work, where we only plotted RDFs to a distance of 6 Å to better compare to the smallest simulation with only 47 waters,^23^ here we present the RDFs to half the box size for all three sets of simulations. Thus, for the largest simulation with 128 waters (red curve), we can capture the hydration structure all the way out to the third solvation shell.

It is noteworthy that there are considerable changes to both the RDF peak heights and locations as the number of water molecules in the system changes, indicating a lack of convergence of the solvent structure with the system size. There are no appreciable differences in the structure of the central cavity as a function of the system size outside the current error bars. When compared to the RDFs of the one-electron MQC models examined by Neupane et al.,^20^ the pair distribution functions generated by our PBEh-D3 simulations (as well as those seen in DFT-based simulations from other groups^21,22,36^) are far more locally structured.^29^

**Figure 2:**
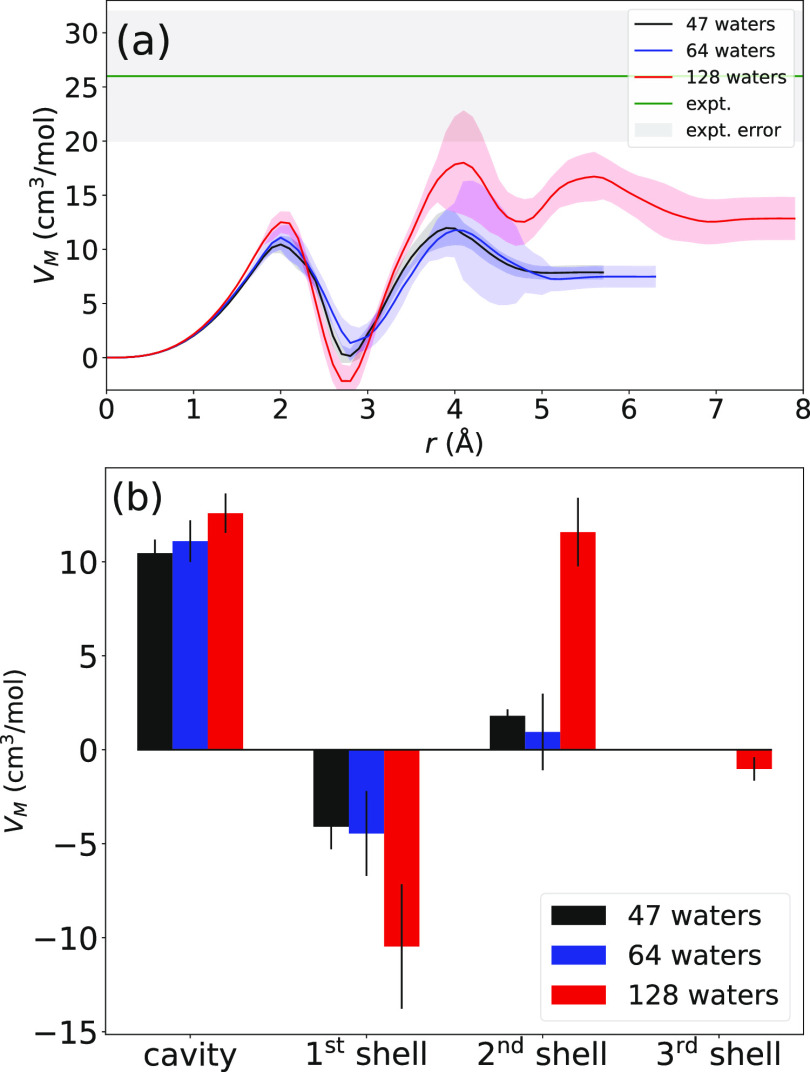
(a) DFT-based ab initio hydrated electron partial molar volume as a running K–B integral. Error bars were computed using block averaging, as described in the Supporting Information. The total integrated partial solvation volumes are 7.87, 7.48 and 12.9 cm^3^/mol for the 47-, 64-, and 128-water simulations, respectively. (b) System size dependence of the cavity and first-, second- and third-shell contributions for the DFT-based solvated electron partial molar volume, again following Neupane et al.^20^ The end-point of the cavity region was taken as the distance where the RDF first reached a value of unity, and the various shell regions were integrated between the corresponding RDF minima. The third shell is defined only for the 128-water system due to box-size limitations with the smaller simulations. Clearly, neither any of the contributions nor the total integration are converged with respect to system size.^23^ The green line and shaded region at the top show the experimental value and uncertainty. The fact that the simulations disagree with experiment by a factor of 2 to about 3.5 depending on system size indicates that the RDF seen in Figure 1 (and in other DFT-based hydrated electron simulations^22,36^) cannot be correct; we have previously argued that DFT simulations with system sizes and functionals similar to those used here produce a strongly overstructured hydrated electron.^7,23,29^

Figure 2a shows the running integral of the partial molar volumes of the DFT-simulated hydrated electron at each of the three system sizes. At the longest distances available based on the simulated system size, the solvation volumes are 7.9 cm^3^/mol for the 47-water system (black curve), 7.5 cm^3^/mol for the 64-water system (blue curve), and 12.9 cm^3^/mol for the 128-water system (red curve). We note that the way the solvation volume changes with system size is nonmonotonic, which we believe is a signature of the fact that the system properties are not converged, even with 128 waters. And although none of the simulations predict a partial molar volume that is within the experimental uncertainty (light green shaded region at the top of Figure 2a), we re-emphasize that at the 64-water size used by other groups,^22^ the predicted solvation volume is off from experiment by a factor of ∼3.5. Since the *V*_M_ is directly calculated from the RDF, there must be aspects of the RDF predicted by DFT, at least with the currently examined functionals and system sizes, which are incorrect. Our previous work^23^ has examined aspects of the DFT electron hydration structure such as coordination number and water dipole angular distributions,^29^ which are also subject to convergence issues with small system sizes, but there are no experimental observables with which to compare these predicted quantities.

Figure 2b shows the contributions of the different solvation regions to the calculated e_hyd_^–^ partial solvation volume. Following Neupane et al.,^20^ we determined the cavity region contribution by integrating the K–B integral until the RDF first reaches a value of unity. We then determined the different solvation shell contributions by integrating the RDF between the minima that separate the various shells. The cavity and first-shell contributions are the same within error at the 95% confidence interval; however, the second shell contribution shows a large increase for the 128-water system due to narrowing of this solvent shell’s peak in the RDF, again suggesting that the simulations are not fully converged with respect to the system size.

Due to the limited box size, we were unable to calculate a third-shell contribution to the partial solvation volume of the hydrated electron for the 47- and 64-water simulations; however, we do find a small negative partial molar volume contribution (*V*_M_ ∼−1 cm^3^/mol) for the third shell of the 128-water DFT-based simulation. It is also interesting to note that, unlike the one-electron systems examined by Neupane et al.,^20^ the second-shell contributions to the partial solvation volume for the DFT-simulated electron are positive, reflecting how generally narrow the second-shell peak in the RDF is at all system sizes.

As mentioned in the Methods section, it is important to consider the way the K–B integral converges for the DFT hydrated electron, given the short trajectory times and small box sizes accessible to AIMD simulations. Indeed, without the weighting function, studies have suggested that hundreds of nanoseconds are required for the K–B integrals to converge, at least in aqueous mixtures of methanol and urea.^51^ This is why in our calculations of *V*_M_, we (as well as Neupane et al.^20^) apply a weighting function to account for the transition from an infinite to finite integral of the RDF, which great improves the convergence.^20,40^ Because it is computationally infeasible to test the convergence of the K–B integrals for the DFT hydrated electron with hundreds of water molecules on nanosecond time scales, we instead tested the convergence on an ion with a similar solvent structure as the DFT hydrated electron, aqueous chloride. In the Supporting Information, we present results for simulated aqueous chloride with 47, 64, and 128 water molecules and a 20 ps sampling time, the same as for our AIMD hydrated electron simulations. We find that the K–B calculated *V*_M_ of Cl^–^ in these simulations is slightly underestimated compared to the value obtained with larger simulation cells and longer simulation times,^52^ and that the value is not fully converged at the 128-water simulation size. However, all three simulations are able to predict the “true” *V*_M_ within 10%, indicating that small simulations like those accessible with AIMD do provide a reasonable estimate of the *V*_M_ at larger system sizes and longer simulation times. Thus, our calculation of the DFT hydrated electron’s *V*_M_ should at least serve as a reasonable estimate of the converged value, meaning that even if our simulations underestimate the true value by ∼10%, they still predict a *V*_M_ that disagrees with experiment by a factor of 2 to 3, depending on the simulation size.

We note that it is not possible to determine exactly what kinds of changes to the RDF are necessary to produce the experimental value of *V*_M_. One possibility is that minor differences in the second and third solvation shells could help bring the DFT-predicted *V*_M_ closer to experiment, as these are weighted by a factor of *r*^2^. However, our analysis on the system size dependence of the *V*_M_ of aqueous chloride (see the Supporting Information) shows that contributions of the third solvation shell to *V*_M_ are also small (∼1 cm^3^/mol). Given the similarity of the chloride and DFT-simulated hydrated electron RDFs, it appears unlikely that higher-shell contributions could account for the factor of ∼2 difference between our 128-water prediction and the experimental value of the *V*_M_ of the hydrated electron. This suggests that significant changes to the size of the central cavity and tightness of the first solvent shell are necessary to correctly predict the experimental *V*_M_ of the hydrated electron. Indeed, the Turi–Borgis (TB) model of the hydrated electron has a qualitatively different RDF than that predicted by DFT, with a larger central cavity and much more poorly defined first shell, and it has a calculated *V*_M_ closer to the experimental value.^20^

**Figure 3:**
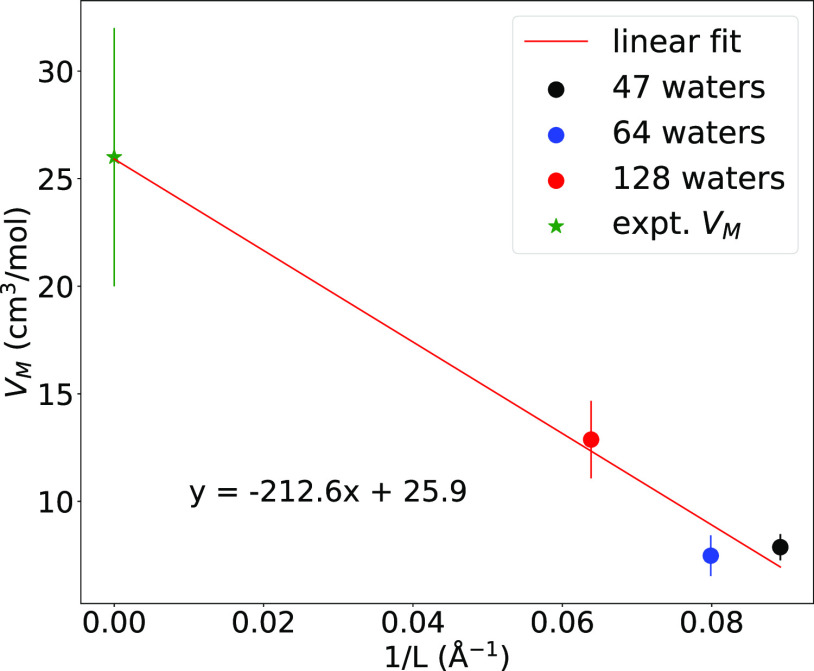
Partial molar volume of the ab initio DFT-simulated hydrated electron plotted against the inverse of the simulation box size, with the experimental value plotted at the infinite box size limit (green star). A linear extrapolation fit to the three simulation sizes yields a *y*-intercept value of ∼26 cm^3^/mol, which agrees with the experimental value.^11,12^ Despite this agreement with experiment, the trend in *V*_M_ is not monotonic with system size, and the value predicted from the 128-water simulation is still off from experiment by a factor of 2, indicating that even the RDF generated by the 128-water simulation must be incorrect.

Finally, Figure 3 shows the partial molar volume plotted against the inverse of the simulation box length. A linear fit and extrapolation to infinite box size yields a partial molar volume of ∼26 cm^3^/mol, which agrees with experiment, despite the fact that the 128-water simulation yields a partial molar volume that is only about half that of experiment. This extrapolation could suggest that DFT calculations using a much larger simulation box with thousands of water molecules might produce the correct hydration structure of the hydrated electron, but it is also possible that the nonmonotonic behavior of the calculated *V*_M_’s with system size indicates that the extrapolated agreement with experiment is fortuitous. At this stage, we cannot state with certainty whether the mismatch between the 128-water simulations and the experimental value is due to the finite system size, the use of DFT, or some combination of both, but the fact that no DFT-based simulations at any tractable system size can predict *V*_M_ correctly to within a factor of 2 of experiment indicates that there are aspects of the DFT-predicted RDFs that disagree with experiment. The MQC TB model and a recent “soft-cavity” MQC model optimized to reproduce the electron’s experimental radius of gyration and eigenvalue both produce a qualitatively different structure than DFT with *V*_M_’s that are closer to experiment.^17^ It remains to be seen whether larger simulation sizes or the use of different exchange–correlation functionals with DFT can predict a solvation structure with the correct *V*_M_.

## 4 Conclusions

In summary, we have extended the K–B formalism for calculating the partial solvation volume of the hydrated electron used by Neupane et al.^20^ to a DFT-based model of the hydrated electron at different simulation sizes. We find that DFT simulations with the PBEh-D3 functional with up to 128 waters yield a *V*_M_ that is significantly too small compared to experiment, a direct reflection that there are aspects of the DFT-predicted solvation structure of the e_hyd_^–^ that must be incorrect.^29^ Thus, *V*_M_ is an experimental observable that DFT simulations have been unable to correctly predict for the hydrated electron,^23,29^ at least at the system sizes presented here and with hybrid exchange–correlation functionals such as PBEh-D3. Given the intimate relationship between the partial molar volume and the RDF of the hydrated electron, these results indicate that the e_hyd_^–^ RDF produced by current computationally feasible DFT simulations is incorrect. However, until we have additional means to compare theoretical predictions of e_hyd_^–^ structure to experimental observables, it remains to be seen whether this discrepancy is due to quantitative differences from the further solvation shells that cannot be simulated with small system sizes or to qualitative differences in the shape of the cavity and first solvent shells in the RDF. To date, however, the model that has produced the closest *V*_M_ to the experimental value is the TB model,^20^ which has a qualitatively different RDF with a larger central cavity and a much less defined first solvation shell than that predicted by DFT.

We note that our results do not preclude the possibility that DFT-based simulations using far larger simulation sizes or different exchange–correlation functionals (particularly functionals that are known to better reproduce the behavior of liquid water^53,54^), could produce a different hydration structure that yields a partial molar volume closer to experiment. It is worth noting, however, that of necessity a simulation that predicts a *V*_M_ that disagrees with experiment must have an incorrect solvation structure to some degree, but a simulation that achieves a correct value of *V*_M_ does not necessarily guarantee that the predicted hydration structure is correct. This is because it is possible for a simulation to have the correct molar solvation volume by coincidence but also fail to explain many other experimental properties of the hydrated electron. For example, the TB MQC model examined by Neupane et al. does give a *V*_M_ that agrees well with experiment, suggesting that its hydration structure is closer to the true structure than the DFT simulations presented here, at least from the *V*_M_ metric. However, the TB model’s solvation structure is likely also incorrect, as it shows no temperature dependence,^2,4^ predicts an inhomogeneously broadened absorption spectrum,^28^ and predicts time-resolved photoelectron spectroscopy dynamics that are all in contrast with experiment.^6^ We close by noting that the formalism introduced by Schnell et al., refined by Krüger and Vlugt,^40,41^ and used by Neupane et al.^20^ is an important step forward because of the ease with which it allows the partial molar volume to be both calculated and interpreted from any hydrated electron simulation, which should greatly increase the ability to make contact between theory and experiment in terms of the structure of this fascinating object.

## Supporting Information Available

Details on uncertainty calculation and error bars; analysis of other sources of uncertainty or error; analysis of convergence for RDFs (PDF)
