# Supplementary material for: Partial Molar Solvation Volume of the Hydrated Electron Simulated Via DFT
Source: J Phys Chem B. 2024 Feb 29;128(10):2425–31. doi: 10.1021/acs.jpcb.3c05091 (PMC10945486; doi:10.1021/acs.jpcb.3c05091)
Supplement: Supplementary file 1 — jp3c05091_si_001.pdf [file jp3c05091_si_001.pdf]

Supporting Information for "The Partial Molar Solvation Volume of the Hydrated Electron Simulated via DFT"

William R. Borrelli,[†,‡] Kenneth J. Mei,[†,‡] Sanghyun J. Park,[†] and Benjamin J. Schwartz[*,†]

†*Department of Chemistry and Biochemistry*

University of California, Los Angeles

Los Angeles California, 90095-1569, United States

‡*Contributed equally to this work*

E-mail: schwartz@chem.ucla.edu

Contents

Uncertainty Calculation **2**

Comparison of Uncertainty Measures 4

Other Sources of Uncertainty or Error **7**

Radial Distribution Function Bin Size Analysis 7

Dependence of Simulation Time on Radial Distribution Function Convergence . . 7

Dependence of the Molar Volume on the Value of the Isothermal Compressibility of Water (κ_T) 7

Classical Chloride Ion Test Case 10

Uncertainty Calculations

Uncertainties for the calculated radial distribution functions (RDFs) and values pertaining to the partial molar volume (V_M) were calculated using block averaging to remove any serial correlations from our time-series molecular dynamics data. All block averaging uncertainty analysis was done using pyblock[1] version 0.4 with Python version 3.8.17. Reported uncertainties were those associated with the optimal block given by pyblock's find_optimal_block() functionality. When an optimal block was not found, the block uncertainty with the largest uncertainty value was reported. The convergence of the standard error as a function of block iteration is plotted in Figure S1. Additional analysis of the uncertainty using bootstrapping, shown in Figure S3, is done using the SciPy package,[2] with $N = 1000$ re-samples.

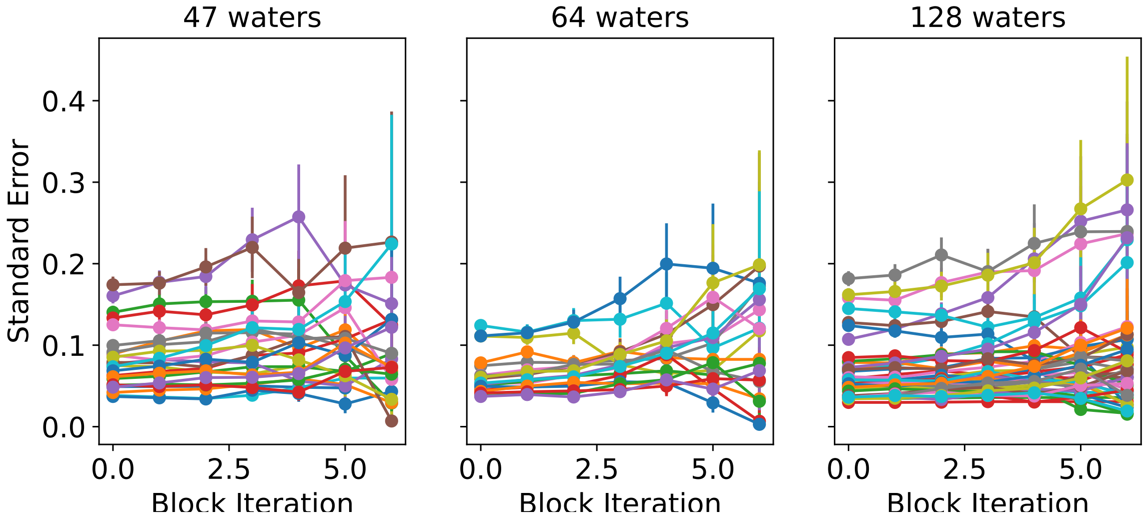

Figure S1: Standard error as a function of block iteration for the 47-, 64-, and 128-water DFT-simulated electron–water center of mass radial distribution function bins. Each curve represents the convergence of the standard error for a single bin of the radial distribution function at the respective system size. The optimal block was chosen using pyblock's $find\_optimal\_block$ function. Due to the limited amount of statistics available to AIMD simulations, the standard errors do not plateau for certain bins and thus serve only as a best estimate. Further details are discussed in the "Comparison of Uncertainty Measures" section below.

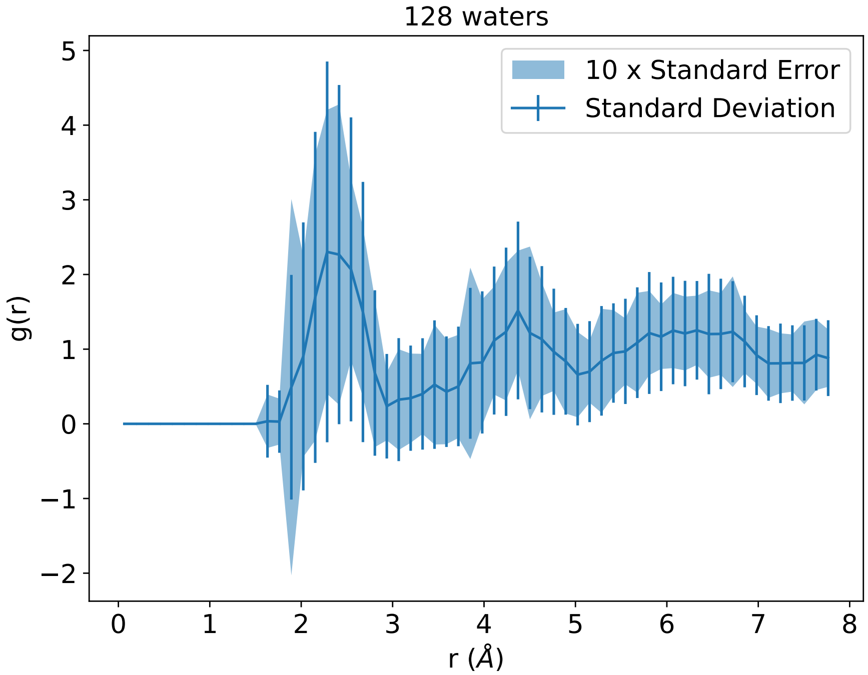

Figure S2: Comparison of possible measures of the uncertainty for the 128-water DFT-simulated electron–water center of mass radial distribution function. The blue shading corresponds to the computed standard errors from block averaging multiplied by a factor of 10 (i.e., five times larger than what is shown in the main text), while the error bars represent the raw standard deviation of each bin. The raw standard deviation serves as the most conservative possible limit on the magnitude of the uncertainties and indicates that our estimated standard errors from block averaging are not underestimated by an order of magnitude, so that the differences between the simulated and experimental V_M's for this system are real outside the error; see also Fig. S4.

Comparison of Uncertainty Measures

Due to the limited statistics available to *ab initio* molecular dynamics (AIMD) simulations, the uncertainties of some bins of our radial distribution function do not plateau in Figure S1, which means these errors could be underestimated. To determine the degree of this possible underestimation, we compare the standard error computed with block averaging, multiplied by a factor of 10, with the raw standard deviation of the counts in each bin (which serves as the upper limit of the possible uncertainty) in Figure S2. This comparison indicates that the computed uncertainties we show in the main text with block averaging are not underestimated by more than an order of magnitude. To explore this further, we provide a comparison of various methods to compute the uncertainty of the radial distribution function in Figure S3. The methods tested include block averaging, bootstrapping, confidence intervals from the Student's t-distribution (the method used by Neupane et al.[3]), the raw standard error, and the raw standard deviation. The uncertainties presented in our main text use the 95% confidence interval from block averaging (top left panel of Fig. S3), the most conservative estimate with the exception of the raw standard deviation. Given the fact that all radial distribution functions of the hydrated electron simulated with AIMD (and ML potential extensions thereof) presented in the literature to date qualitatively resemble the ones presented here, the uncertainties we use on the main text appear to be a quite conservative estimate.

Figure S4 panels (a-d) show the partial molar volume running integrals plotted using 4 different measures of uncertainty. Even with the largest possible choice for the error bars, the predicted V_M's from the DFT-based simulations do not agree with experiment within error.

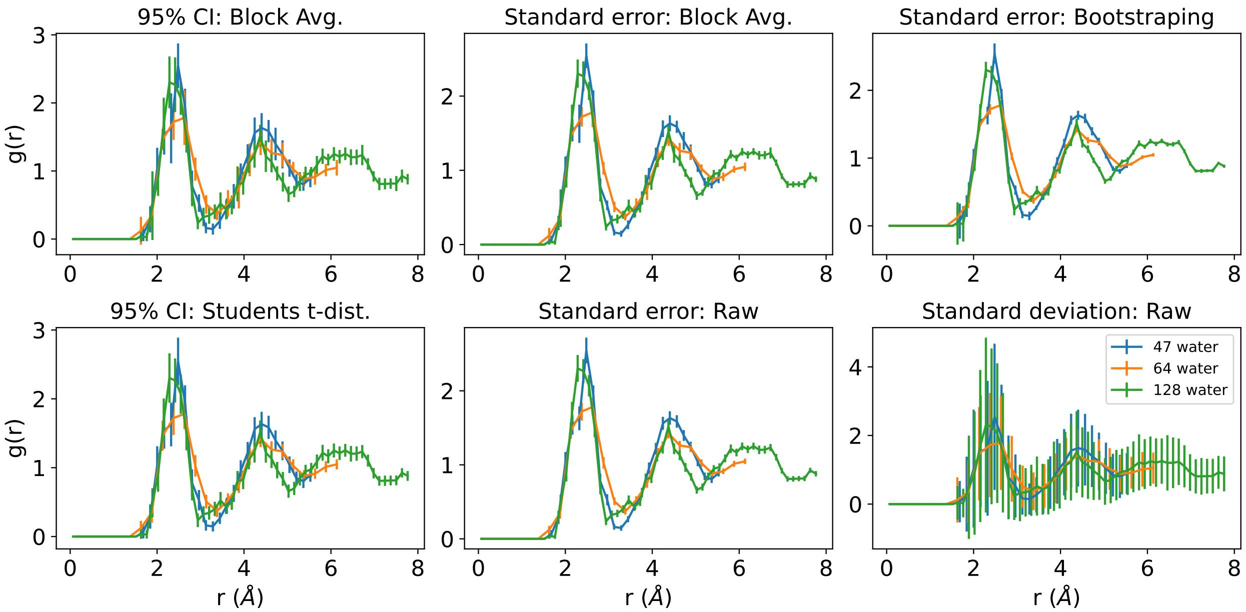

Figure S3: Comparison of different uncertainty calculations of the DFT-simulated electron–water center of mass radial distribution function for each system size studied in this work. The blue, orange, and green curves correspond to the 47-, 64-, and 128-water systems, respectively. Various uncertainty calculations include 95% confidence interval from block averaging (top left), standard error from block averaging (top middle), standard error from bootstrapping (top right), 95% confidence interval from Student's t-distribution (bottom left), raw standard error (bottom middle), and the raw standard deviation (bottom right). The reported radial distribution function uncertainty in the main text uses the 95% confidence interval from block averaging (top left), which yields the most conservative error bars with exception of the raw standard deviation (which clearly overestimates the uncertainty of the radial distribution function).

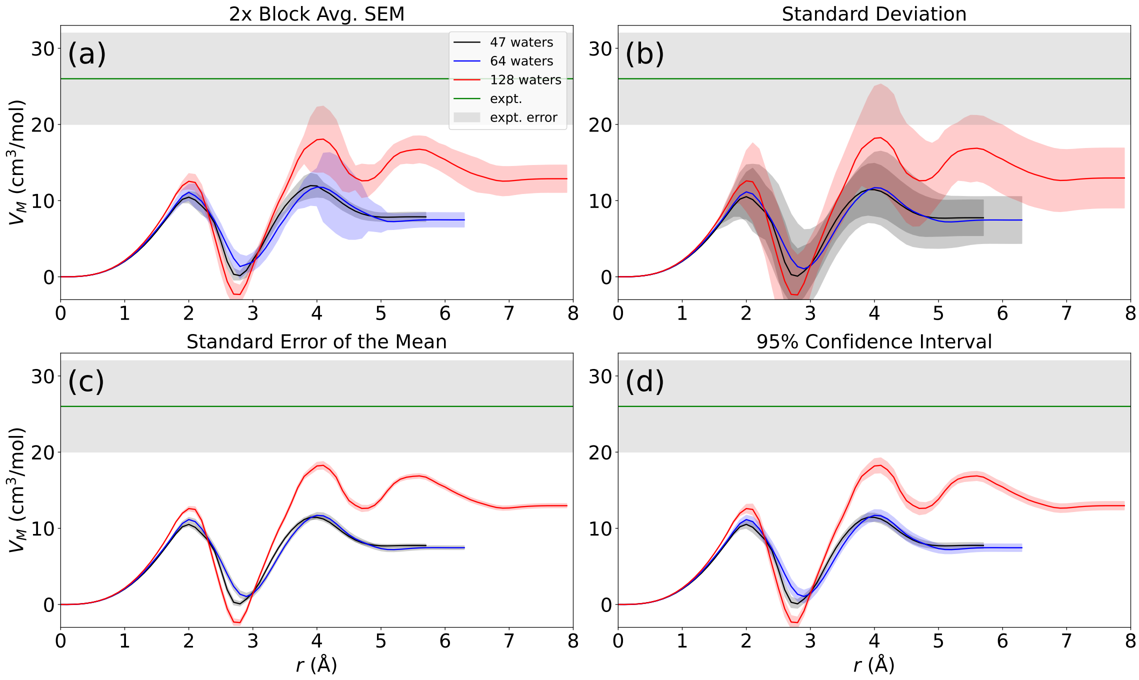

Figure S4: The K-B partial molar volume running integral for DFT-based simulations of the hydrated electron plotted with 4 different measures of uncertainty: (a) block averaging 95% confidence interval, (b) standard deviation over a time average, (c) standard error of the mean over a time average, (d) 95% confidence interval from the t-distribution. Panel (a) was the method presented in the text, as block averaging is a known method for removing possible serial correlation in time series data, and is most pertinent to use given the limited simulation times currently accessible via *ab initio* DFT-based (PBEh-D3) methods. We note that even with the most generous possible uncertainty, panel (c), the calculated V_M for all system sizes does not match experiment within error.

Other Sources of Uncertainty or Error

Radial Distribution Function Bin Size Analysis

Figure S5 shows the impact of bin count on the calculated partial molar volume ($\mathrm{V_M}$). We note that the final value of V_M changed less than 1% for a wide range of bin size choices. For the analysis presented in the main text, we chose to use bin counts of 35, 25, and 60 for the 47-, 64- and 128-water DFT-simulated radial distribution functions (RDFs), respectively, as these values were converged with the predicted V_M and allowed the most statistics in each bin.

Dependence of Simulation Time on Radial Distribution Function Convergence

Since the partial molar volume is quite sensitive to changes in the RDF, we calculated RDF's using different fractions of our simulation data to better judge the convergence. Figure S6 shows RDF's for the DFT-simulated hydrated electron for each system size calculated using 25%, 50%, 75%, and 100 % of our data. The results indicate that the RDFs are converged using only half the simulation data for the 47-water simulation and 75% of the data for the 64- and 128-water simulations. The figures presented in the main text used 100% of the data.

Dependence of the Molar Volume on the Value of the Isothermal Compressibility of Water (κ_T)

Since the K-B integral used to compute V_M from the RDF depends on the isothermal compressibility, it is possible to obtain different values of V_M depending on whether one uses an experimental or simulated value of κ_T. Figure S7 shows the predicted values of V_M for different possible choices of κ_T, including κ_T values from experiment (red bars),[4] simulations

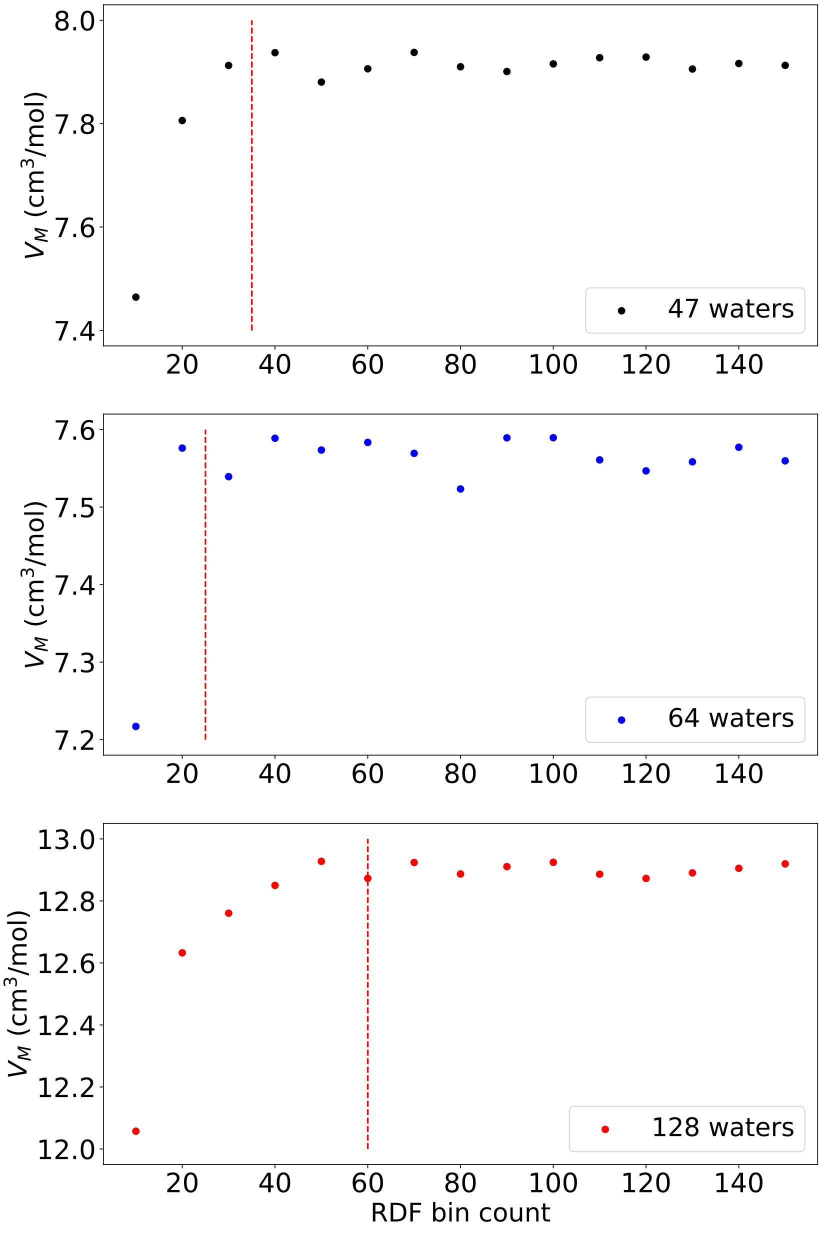

Figure S5: Variation of the predicted partial molar volume of the hydrated electron with the bin count for the 47-, 64-, and 128-water DFT-based simulations. The number of bins used in our RDF and the calculated V_M are 35, 25, and 60 for the 47, 64, and 128 water simulations, respectively. The data show that our chosen bin size is adequate for all simulation sizes and that changing the RDF bin size has a negligible impact on the calculated partial molar volume (on the order of 0.01-0.04 cm^3/mol).

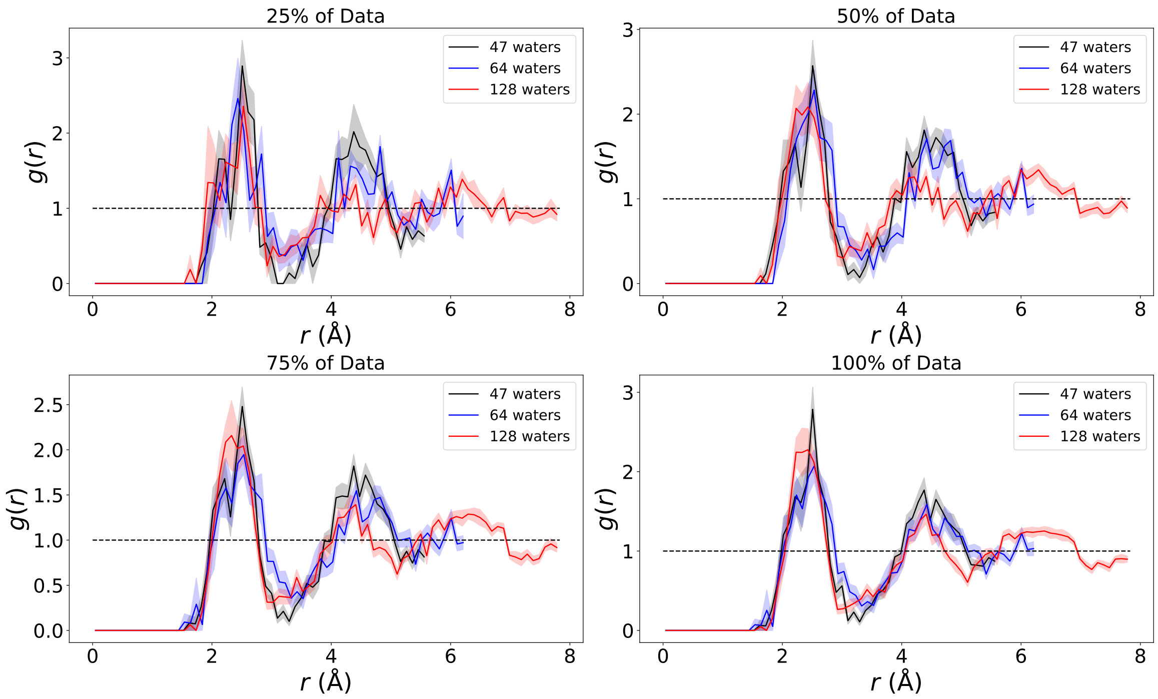

Figure S6: RDFs of the DFT-simulated hydrated electron for all 3 simulation sizes as a function of the fraction of the available trajectory data used to compute the RDF. The 47-water simulation is converged using only 50% of the available data, while the 64- and 128-water simulations are clearly converged using only 75% of the data; 100% of the data was used in the main text.

of SPC/Fw water,[5] which is the value used by Neupane et al.[3] (yellow bars), DFT-based simulations of liquid water using the PBEh+D3 level of theory (green bars),[4] and simulations of liquid water using the PBEh+D3 level of theory with temperature correction (blue bars).[4] The choice of κ_T makes only a subtle difference in the predicted V_M outside the error bars, and in the main text, we used the PBEh+D with temperature correction value as appropriate to match our simulations of the hydrated electron. We note that the difference in the calculated V_M between the PBEh+D and PBEh+D with temperature correction κ_T values was negligible.

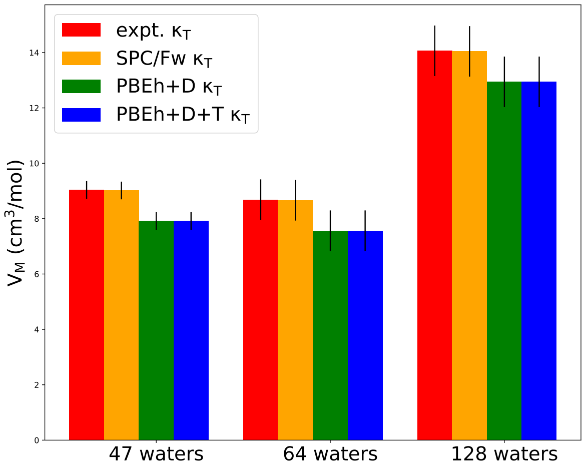

Figure S7: V_M of the DFT-simulated hydrated calculated for each system size using either the experimental, SPC/Fw, PBEh+dispersion correction, or PBEh+dispersion+temperature correction κ_T values.

Testing the Convergence of V_M Calculated via the K-B Method with a Classical Aqueous Chloride Ion

To validate our work for a test case where both the experimental and simulated V_M is known, we simulated a classical chloride ion with 47, 64, and 128 waters and calculated the partial molar volume using the same method we used for the hydrated electron in the main text. This test case is especially useful as the chloride ion is an anion with a very similar RDF to

that of the DFT-simulated hydrated electron (i.e., with highly structured solvent shells with similar peak heights and widths). Moreover, the V_M of chloride is experimentally known to be 23.7 cm^3/mol, while the converged simulation V_M for the particular model we chose is 21.2 cm^3/mol.[6] These values are of the same sign and magnitude as the (experimental) hydrated electron V_M. This provides an excellent opportunity to validate that our analysis (using the same system sizes and trajectory lengths) correctly predicts V_M for a similar anion to the DFT-simulated hydrated electron.

The classical Lennard-Jones parameters for Cl^- that we chose are from the work of Imai et al.,[6] which predict a V_M of chloride that is close to its experimental value. The water model we used in these simulation is SPC/fw with a 0.5-fs time step. We ran these simulations in the N, V, T ensemble at 298 K with box sizes, numbers of water and trajectory lengths chosen to match the AIMD simulation parameters for the hydrated electron. What follows is a reproduction of the analysis done in the manuscript for the hydrated electron but on these additional aqueous chloride simulations.

Figure S8 shows the K-B-calculated V_M values for our simulated aqueous Cl^- as a function of simulation size, with the dashed horizontal line indicating the fully converged simulation value from Imai et al.[6] These results indicate that the systems sizes and trajectory lengths used in our simulations are indeed sufficient to give a reasonable estimate of the true partial molar volume.

Figure S9 shows the chloride-water RDFs for all system sizes. There is an increase in first solvent shell peak intensity going from 47 to 64 waters, along with a shift in peak location for the first and second solvent shells. From 64 to 128 waters there is little change in the first solvent shell peak, but there is a shift in second solvent shell peak location.

Figure S10 shows the different solvent shell contributions to the partial molar volume of aqueous chloride, defined in the same way as in the main text for the hydrated eletron. The cavity contribution shows a monotonic increase with system size that is outside the error, while the first shell contribution shows a monotonic decrease of comparable magnitude. The

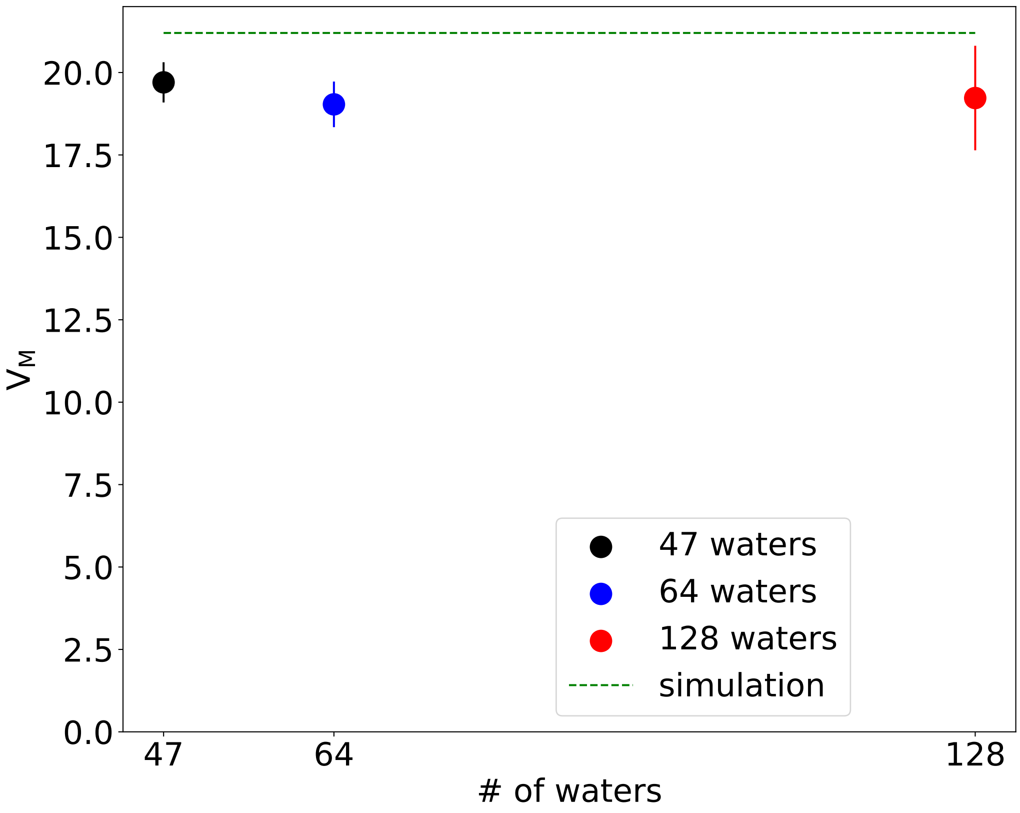

Figure S8: K-B-calculated partial molar volumes for chloride simulated with 47 (black), 64 (blue), and 128 (red) water molecules using the same trajectory lengths as used for our DFT simulations of the hydrated electron. The dashed green line indicates the converged simulation value for V_M.[6] The partial molar volume values for each system size are the same within error, indicating a convergence of this quantity with respect to the number of waters. All of the values are slightly smaller than the fully converged simulation value, although within 10%, and the 128-water value is nearly within error of the converged value.

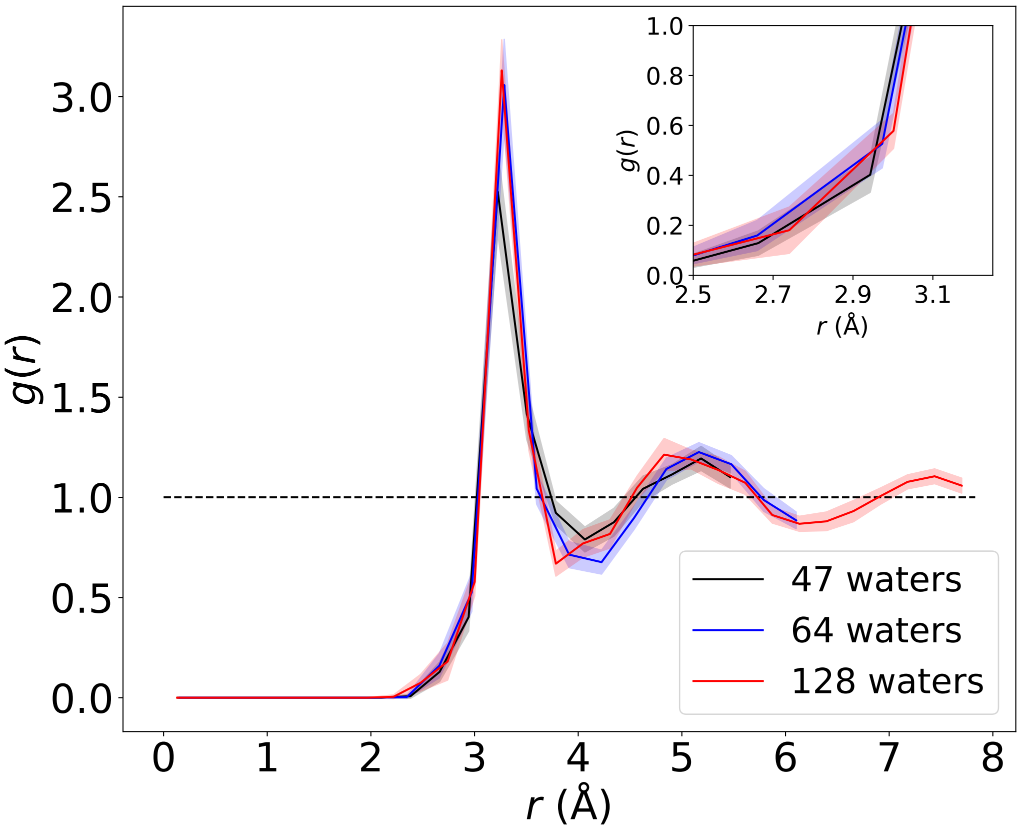

Figure S9: Chloride-water RDFs for the 47-, 64-, and 128-water simulations. Going from 47 to 64 waters causes a change in first solvent shell peak height as well as slight shifts in the first and second solvent shell peak locations. From 64 to 126 waters there is little change in first solvent shell peak height, however, there is a shift in second solvent shell peak location. These changes are qualitatively comparable to those we observed in the DFT simulations of the hydrated electron, indicating comparable convergence.

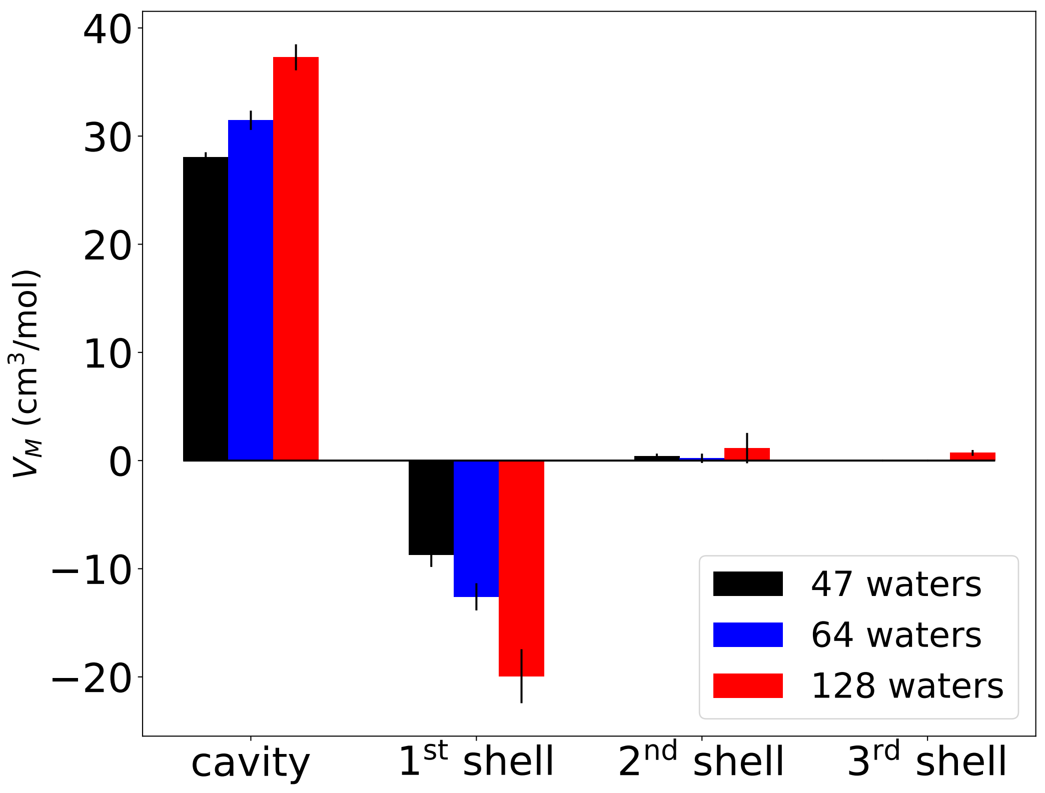

Figure S10: Different solvent shell contributions to the K-B-calculated partial molar volume of chloride, defined in the same way as in the main text for the hydrated electron. The cavity contribution increases monotonically with system size just outside of the error, while the first solvent shell contribution decreases monotonically with system size by a similar amount. The second shell contributions are within error of each other and the third shell contribution (only present for the 128-water simulation) is quite small.

second shell contributions are all within error of each other, and the third shell contribution (only present in the 128 water simulation), like that of the DFT-simulated hydrated electron, is quite small.

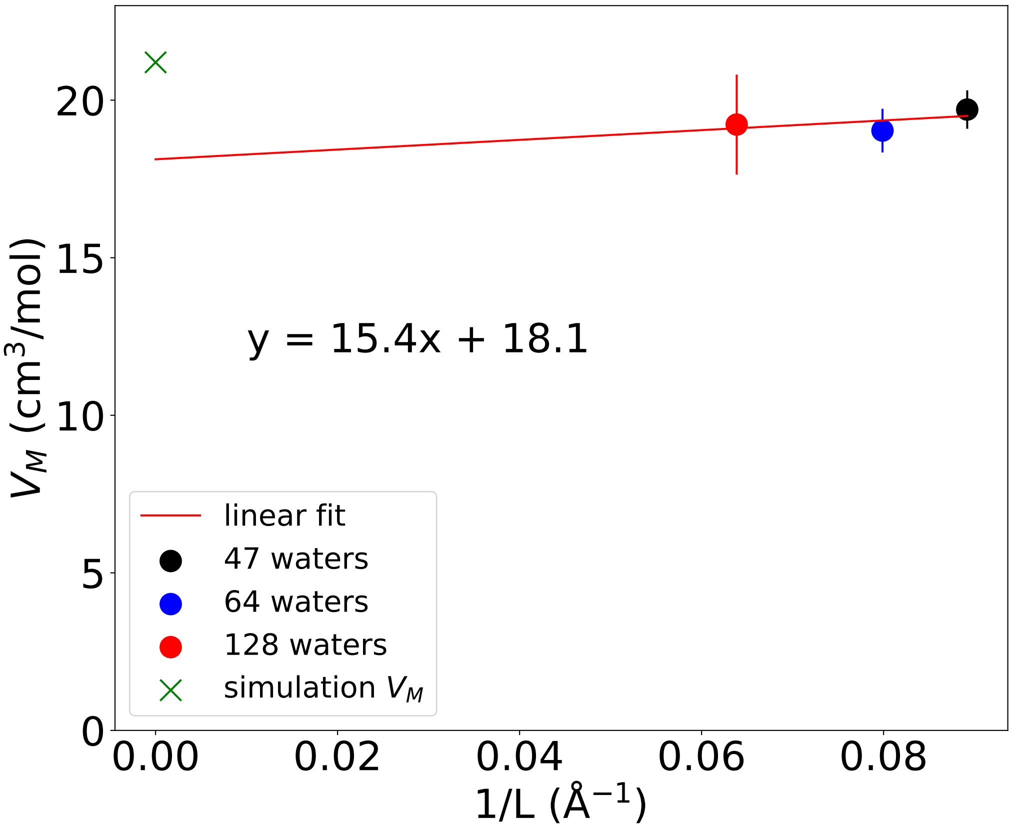

Figure S11: Extrapolation of the partial molar volume of aqueous Cl^- with the inverse of the simulation cell length. The small slope, along with a y-intercept value that is close that of the converged simulation V_M, indicates that the partial molar volume is relatively converged at these system sizes. As with the hydrated electron, the calculated V_M's are not monotonic with system size.

Figure S11 shows an extrapolation of the calculated partial molar volume with the inverse of the simulation cell length for aqueous Cl^-, giving an estimate of what the partial molar volume should be at infinite system size. The relatively small slope of the fit line, as well as the close correspondence of our simulated V_M values to the converged simulated value, indicates that the K-B-calculated V_M is fairly converged at these system sizes. This analysis

also highlights the difficulty in extrapolating non-monotonic data (as was also the case for the DFT-simulated hydrated electron); although the y-intercept predicts a value that is fairly close to the true converged value, the slope has the incorrect sign.

From these test case simulations on the chloride ion and the resulting analyses presented above, it follows that our calculation of the hydrated electron V_M at the present system sizes should give a reasonable estimate to the true simulated DFT V_M for this object.

References

(1) Spencer, J. pyblock. `http://github.com/jsspencer/pyblock`.

(2) Virtanen, P. et al. SciPy 1.0: Fundamental Algorithms for Scientific Computing in Python. *Nature Methods* **2020**, *17*, 261–272.

(3) Neupane, P.; Bartels, D. M.; Thompson, W. H. Relation between the Hydrated Electron Solvation Structure and Its Partial Molar Volume. *The Journal of Physical Chemistry B* **2023**, *127*, 5941–5947, PMID: 37345987.

(4) Gaiduk, A. P.; Gygi, F.; Galli, G. Density and Compressibility of Liquid Water and Ice from First-Principles Simulations with Hybrid Functionals. *The Journal of Physical Chemistry Letters* **2015**, *6*, 2902–2908.

(5) Wu, Y.; Tepper, H. L.; Voth, G. A. Flexible simple point-charge water model with improved liquid-state properties. *The Journal of Chemical Physics* **2006**, *124*, 024503.

(6) Imai, T.; Nomura, H.; Kinoshita, M.; Hirata, F. Partial molar volume and compressibility of alkali-halide ions in aqueous solution: Hydration shell analysis with an integral equation theory of molecular liquids. *Journal of Physical Chemistry B* **2002**, *106*, 7308–7314.
